# Influence of Storage Conditions on Decellularized Porcine Conjunctiva

**DOI:** 10.3390/bioengineering10030350

**Published:** 2023-03-11

**Authors:** Adam Skornia, Gerd Geerling, Kristina Spaniol, Joana Witt

**Affiliations:** Department of Ophthalmology, University Hospital Duesseldorf, Heinrich Heine University, 40225 Duesseldorf, Germany

**Keywords:** conjunctival reconstruction, decellularization, decellularized conjunctiva, scaffold, human conjunctival epithelial cell

## Abstract

Porcine decellularized conjunctiva (PDC) represents a promising alternative source for conjunctival reconstruction. Methods of its re-epithelialization in vitro with primary human conjunctival epithelial cells (HCEC) have already been established. However, a long-term storage method is required for a simplified clinical use of PDC. This study investigates the influence of several storage variants on PDC. PDC were stored in (1) phosphate-buffered saline solution (PBS) at 4 °C, (2) in glycerol-containing epithelial cell medium (EM/gly) at −80 °C and (3) in dimethyl sulfoxide-containing epithelial cell medium (EM/DMSO) at −196 °C in liquid nitrogen for two and six months, respectively. Fresh PDC served as control. Histological structure, biomechanical parameters, the content of collagen and elastin and the potential of re-epithelialization with primary HCEC under cultivation for 14 days were compared (n = 4–10). In all groups, PDC showed a well-preserved extracellular matrix without structural disruptions and with comparable fiber density (*p* ≥ 0.74). Collagen and elastin content were not significantly different between the groups (*p* ≥ 0.18; *p* ≥ 0.13, respectively). With the exception of the significantly reduced tensile strength of PDC after storage at −196 °C in EM/DMSO for six months (0.46 ± 0.21 MPa, *p* = 0.02), no differences were seen regarding the elastic modulus, tensile strength and extensibility compared to control (0.87 ± 0.25 MPa; *p* ≥ 0.06). The mean values of the epithelialized PDC surface ranged from 51.9 ± 8.8% (−196 °C) to 78.3 ± 4.4% (−80 °C) and did not differ significantly (*p* ≥ 0.35). In conclusion, all examined storage methods were suitable for storing PDC for at least six months. All PDC were able to re-epithelialize, which rules out cytotoxic influences of the storage conditions and suggests preserved biocompatibility for in vivo application.

## 1. Introduction

Together with the cornea, the conjunctiva is a functionally significant part of the ocular surface. In histology, it appears as a multi-layered, non-keratinized squamous epithelium with secretory goblet cells and a loose connective tissue underneath. Its main functions are based on shielding the surface of the eye, contributing to the tear film, immunological defense and enabling eye motility. Pathological changes through chemical burns, exposure to heat, autoimmune diseases, neoplasia or mechanical trauma can result in significant damage to the conjunctiva, leading to insufficiency or a reduction in local stem cells and causing squamous metaplasia and conjunctival scarring [1]. In severe cases, surgery with tissue replacement is required [2].

Commonly used graft tissues such as human amniotic membrane or autologous mucous membrane have certain drawbacks, including limited availability, time-consuming preparation, and inconsistent tissue properties such as thickness or growth factor content, which have led to an increased focus on alternative tissues and materials [3,4,5,6,7,8]. In this context, extracellular matrix-based biomaterials and decellularized tissues are becoming more important and have already been used clinically [9,10,11,12,13,14,15]. The biochemical composition of the extracellular matrix is unique for every organ and has an effect on specific tissue properties and cell–matrix interactions [16,17,18]. Removing cells from tissues and even entire organs through physical, chemical and biological processes creates acellular scaffolds that otherwise closely resemble the tissue that has to be replaced [19,20]. The lack of immunogenicity and the biochemical and biomechanical characteristics similar to the original tissue promise high biocompatibility and potentially enhance regeneration [9,21].

We have developed a protocol to decellularize the conjunctiva from pigs (*Sus scrofa domesticus*). Decellularized porcine conjunctiva (PDC) maintains its tissue properties compared to the native conjunctiva and shows high biocompatibility in vivo [22]. Methods of re-epithelialization of PDC in vitro with primary human conjunctival epithelial cells (HCEC) have already been established and tested in a rabbit model showing preserved progenitor cell properties and superior tissue repair compared to amniotic membrane [23]. Another research group already described the clinical application of PDC in four patients, three with pterygium and one with symblepharon. The grafts in all cases were completely re-epithelized and no graft melt or fibroplasia were observed [11].

Despite the increasing interest in decellularized tissues in modern medicine, one hurdle to their clinical application is the lack of standardized long-term preservation methods and the limited off-the-shelf availability. Requirements for a suitable storage method are the preservation of tissue architecture, biochemical composition and preservation of biomechanical properties. There is no general recommendation for storage of decellularized tissues as the effects of the same storage method on different tissues may vary.

Few studies have been performed on the storage of decellularized tissues. Cryopreservation is the most commonly used method in tissue banking [24]. This technique is based on tissue storage in cell medium with addition of cryoprotectants such as dimethyl sulfoxide (DMSO) or glycerol [25]. In particular, controlled-rate freezing (−1 °C/min) in liquid nitrogen at −196 °C with DMSO is a gold standard within cryopreservation and shows good results regarding the ECM preservation of decellularized matrices [26,27,28].

Additionally, storage at −80 °C in glycerol-containing freezing medium (cell medium + glycerol 85%, 1:1 *v*/*v*) is a currently widespread storage method for amniotic membrane, the most frequently used tissue for conjunctival reconstruction [29,30]. Another storage method commonly described is refrigeration in phosphate-buffered saline (PBS) at 4 °C. PBS is widely used in laboratories and the pH of the medium corresponds to the physiological pH of human cells. As several studies reported different results with regard to storage time, PBS is primarily considered as a short-term storage option [26,27,31,32].

The aim of this study was to evaluate the effect of these established storage methods on PDC.

## 2. Materials and Methods

### 2.1. Materials and Instruments

The specifications of materials, instruments and chemicals used are listed in Table 1.

### 2.2. Preparation and Storage

Porcine eyeballs were provided by a local abattoir and transported in phosphate-buffered saline solution (PBS). The bulbar conjunctiva was dissected from the eyes within 4 h and rinsed with fresh PBS containing 5% penicillin/streptomycin (P/S). The decellularization was carried out as previously described including γ-sterilization with a radiation dose of at least 25 kGy (BBF Sterilisationsservice GmbH, Kernen, Germany) [22]. Afterwards, PDC was stored under three different conditions for two and six months, respectively (Table 2). In the first group, PDC was stored in PBS at 4 °C (=group PBS). In the second group, PDC was transferred to a 1:1 (*v*/*v*) mixture of 85% glycerol (gly) and conjunctival epithelial cell medium (EM) and frozen to −80 °C (=group EM/gly). EM contained Dulbecco’s Modified Eagle’s Medium / F-12 Ham), 10% fetal bovine serum (FBS), 0.4 µg/mL hydrocortisone, 0.01 nM cholera toxin, 0.075% sodium bicarbonate, 0.18 mM adenine, 2 nM triiodo-L-thyronine, 5 µg/mL transferrin, 5 µg/mL insulin, 1% antibiotic–antimycotic solution and 10 ng/mL human EGF. In the third group, PDC was transferred to 80% EM with additional 10% FBS and 10% dimethyl sulfoxide (DMSO). Samples were frozen in freezing containers at a cooling rate of −1 °C/min to −80 °C and transferred to liquid nitrogen (−196 °C) on the next day (=group EM/DMSO). After two and six months, frozen PDC was thawed overnight at 4 °C and washed three times in PBS before testing. Fresh PDC stored in PBS at 4 °C for a maximum of 96 h served as a control.

### 2.3. Histological Analysis

Four PDCs were each divided into seven parts for every storage method and study period, as well as the control to reduce donor variability. For evaluation, PDCs were fixed in 4% paraformaldehyde for 1.5 h, then embedded in paraffin and cut into 4.5 µm slides with a rotary microtome. Afterwards, hematoxylin/eosin staining was performed. For evaluation, three representative images from different section heights were assessed for each PDC. ECM density was measured using ImageJ Version 1.53e [33].

### 2.4. Transmission Electron Microscopy

For analysis of ultrastructure, transmission electron microscopy (TEM) images were taken by the *Core Facility Electron Microscopy* of the Heinrich Heine University Duesseldorf. PDC fragments from three donors (2 × 2 mm) were each divided into four parts, of which one fragment was assessed as control tissue, the other three parts were stored for six months after one of the three methods before analysis. PDC were fixed in a mixture of 4% paraformaldehyde and 2.5% glutaraldehyde in 0.1 M cacodylate buffer at 4 °C for three days. Pure Spurr was used for embedding. Subsequently, ultrathin sections of 70 nm were cut using an ultramicrotome. Images were taken with the transmission electron microscope at an acceleration voltage of 100 kV.

For quantitative analysis of collagen bundle loosening and fiber size, three 1 × 1 µm-sized-cross-sectional areas per donor were analyzed as followed. The number and diameter of collagen fibers were measured using ImageJ. To analyze the interspace between the collagen fibers, the images were made binary. Collagen fibers appeared in white pixels, the interspace in dark pixels. The percentage of dark pixels relative to the total pixel number was compared between the groups.

### 2.5. Content of Extracellular Matrix Proteins

Collagen content (I, II, III, V, XI) was determined by the Sircol™ Insoluble Collagen assay kit. Dry weight in the range of 6.2 (±0.2) mg of PDC was used. The further steps were carried out according to the manufacturer’s protocol. Fastin™ Elastin assay kit was used for the measurement of elastin content following the manufacturer’s protocol.

### 2.6. Biomechanical Properties

Biomechanical evaluation was carried out with the uniaxial material testing machine zwickiLine Z0.5 TN. For this, PDC of a size of 10 × 5 mm were pulled apart until the breaking point. The material was tested with a preload of 0.001 N and a test speed of initially 5 mm/min to determine the elastic modulus (E_mod_) [kPa], for tensile strength [MPa] and the elongation at break in relation to the initial value [%] at a speed of 25 mm/min. Parameters were calculated using the software testXpert III Version 1.5 by ZwickRoell.

### 2.7. Re-Epithelialization

HCEC were obtained by explant culture from human conjunctivas from the Lions Cornea Bank North Rhine Westphalia of the University Eye Hospital Duesseldorf. This study was performed in compliance with the Declaration of Helsinki and was approved by the ethics committee of the Heinrich Heine University Duesseldorf. Written informed consent for research use was obtained from the next of kin of each donor before experiments were performed. HCEC from three different donors were cultivated as described previously [23]. The HCEC were frozen in cryomedium (EM + 10% FBS + 10% DMSO) in freezing containers at a cooling rate of −1 °C/min to −80 °C, transferred to liquid nitrogen the next day and stored until thawing.

For recellularization with primary HCEC, PDCs were clamped into 8.5 mm-diameter cell crowns and placed in six-well culture plates. The PDCs were equilibrated overnight in EM and on the subsequent day, 1 × 10^6^ HCEC per cell crown were seeded onto the PDC. HCEC were cultured at 37 °C and 5% CO_2,_ and the medium was changed every two days. At day seven, the medium was reduced to a level where only the basal side of the cells was in contact with the medium and the top cell layer was exposed to the air (air-liquid-interface cell culture). From this point on, the EGF concentration in the EM was reduced to 0.5 ng/mL. After 14 days of culture, PDCs were fixed for histological analysis as described above.

For evaluation of re-epithelialization capacity, fixed PDC were embedded in paraffin and four sections per PDC were analyzed. The proportion of the epithelialized surface in the total length of the PDC was measured using ImageJ Version 1.53e, [28]. For precise analysis of the epithelial stratification, the percentage of one-, two-, and three- or multi-layered epithelium in the total epithelium was determined.

### 2.8. Statistical Analysis

Statistical evaluation was performed by Prism Version 9.3.1 (GraphPad Software, San Diego, CA, USA) using two-way-ANOVA with Tukey’s test for all data sets except for the evaluation of the re-epithelialization capacity. Here, the Kruskal–Wallis test was chosen as non-parametric test for the unifactorial data set. All values are presented as mean ± standard deviation. The significance level was set at 5%.

## 3. Results

### 3.1. Histological and Ultrastructural Analysis

**Figure 1:**
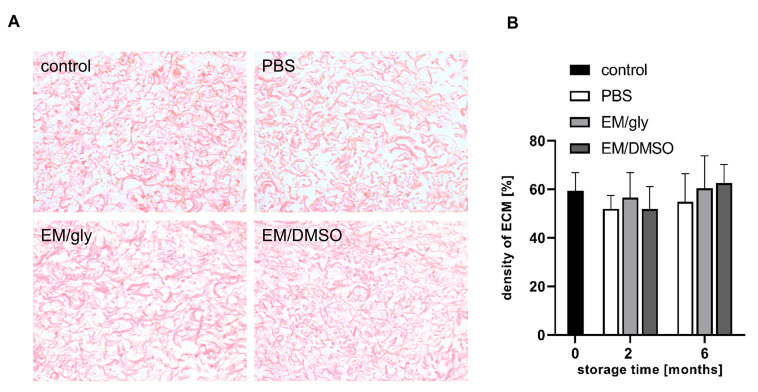
Histological evaluation of fresh PDC (control) and PDC stored in PBS (at 4 °C), in EM/gly (at −80 °C) and in EM/DMSO (at −196 °C). H&E-stained images after storage for six months, scale = 100 µm) (**A**) as well as image analysis (**B**) demonstrated preserved collagen bundles and a maintained ECM density without significant differences between PDC of different storage methods (*p* ≥ 0.74, n = 4).

Histologic cross-sections of PDCs showed comparable density of conjunctival stroma to control and preserved collagen fibers without visible damage or amorphous ECM structure after all storage variations (Figure 1). No storage-induced loosening or destruction of the connective tissue was observed. Quantitative assessment of the images showed no significant differences between groups in terms of ECM loosening. (*p* ≥ 0.74).

**Figure 2:**
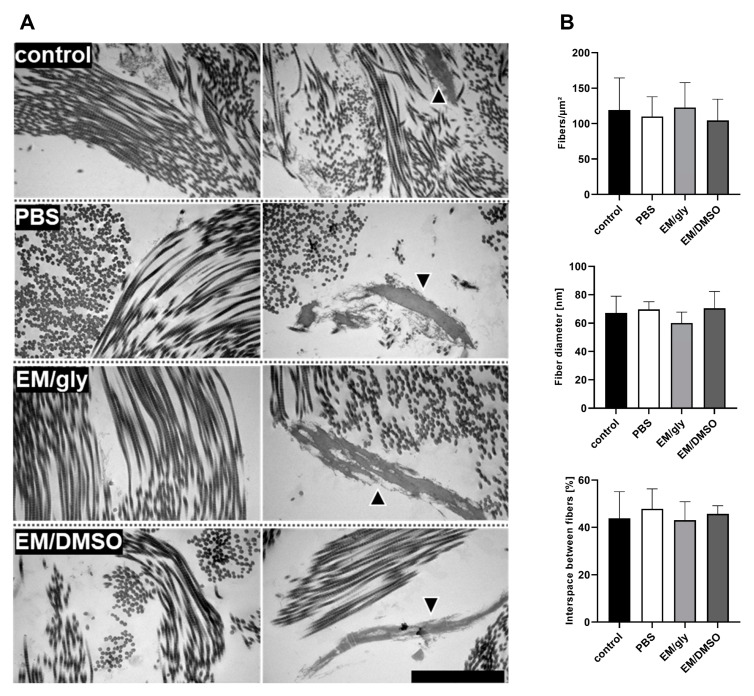
(**A**) Representative TEM images of fresh PDC (control) and PDC stored for six months in PBS (at 4 °C), in EM/gly (at −80 °C) and in EM/DMSO (at −196 °C). The left column shows collagen fibers in longitudinal and cross-sections, and the right column shows images with elastin fibers (arrowheads); scale = 2 µm, n = 3. (**B**) Results of the quantitative analysis of the number of collagen fibers per µm^2^, the fiber diameter, and the space between the fibers.

TEM analysis showed that collagen fiber bundles were intact after all storage variants (Figure 2A). No interruptions in the bundle structures or significant changes in fiber orientation were observed. With regard to fiber boundary and transverse striations, there were no differences between the PDC at higher magnifications in the cross-section.

Quantitative analysis showed that the mean fiber diameter ranged from 60.0 ± 7.8 to 70.5 ± 11.8 nm. No significant difference in fiber diameter was found in any of the storage groups compared to the control group (all *p* > 0.94) or between the storage groups (all *p* > 0.77) (Figure 2B). The number of fibrils per µm^2^ and the space between fibrils were also not significantly different between the four groups (all *p* > 0.89).

Elastin fibers were seen in all groups and appeared as shorter, wider structures (Figure 2A, arrowheads). Differences in the occurrence of elastin were not observed. Elastin structures of both control and PDC stored for six months showed blurred edges within the fiber boundaries and focal reductions in density.

### 3.2. Content of Extracellular Matrix Proteins

**Figure 3:**
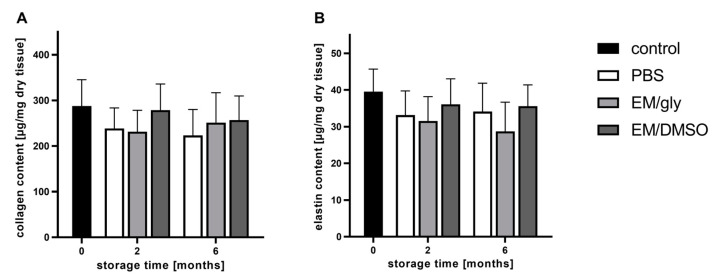
Collagen (**A**) and elastin (**B**) content of fresh PDC (control) and PDC stored for two and six months in PBS (at 4 °C), in EM/gly (at −80 °C) and in EM/DMSO (at −196°C). There were no significant differences between the groups (collagen: *p* ≥ 0.18, elastin *p* ≥ 0.13); collagen: n = 10, elastin n = 6.

Statistical evaluation revealed no significant differences in collagen or elastin content between groups (collagen: *p* ≥ 0.18; elastin: *p* ≥ 0.13; Figure 3) after two or six months of storage. The lowest collagen value was found after six months storage in PBS at 4 °C and the two highest values after two and six months of EM/DMSO storage. The highest mean values for elastin were obtained after EM/DMSO storage and the lowest after EM/gly storage. PDC in the EM/gly group after six months contained the lowest elastin content (28.73 ± 7.27 vs. 39.55 ± 5.62 µg/mg in the control group).

### 3.3. Biomechanical Properties

**Figure 4:**
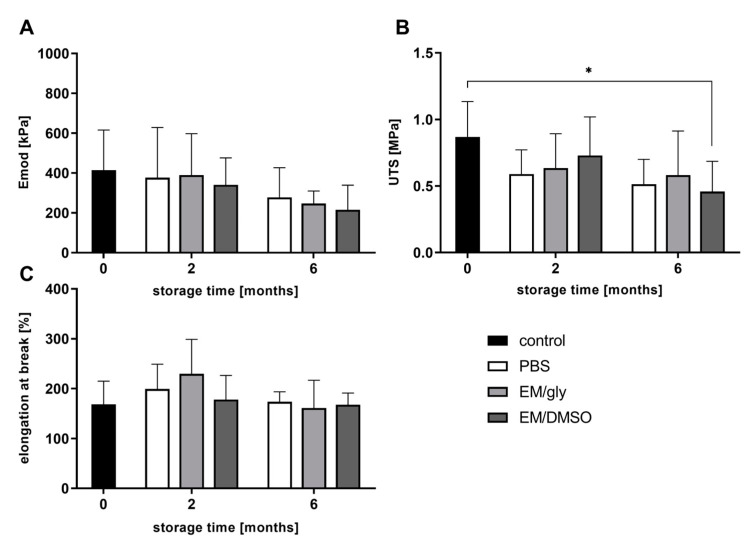
Biomechanical properties of fresh PDC (control) and PDC stored for two and six months in PBS (at 4 °C), in EM/gly (at −80 °C) and in EM/DMSO (at −196 °C). While the elastic modulus (**A**) (*p* ≥ 0.26) and the elongation at break (**B**) (*p* ≥ 0.06) did not show any significant differences, there was a significant reduction in tensile strength (**C**) in the EM/DMSO group compared with the control group after six months (* *p* = 0.02); n = 9.

Biomechanical testing did not show statistically significant differences of E_mod_ between all groups (*p* ≥ 0.26). However, mean values for E_mod_ were reduced after long-term storage (control: 414.14 ± 189.64 kPa; after two months PBS: 376.49 ± 237.18 kPa, EM/Gly: 389.18 ± 196.39, EM/DMSO: 340.74 ± 127.32 kPa; after six months PBS 277.93 ± 140.32 kPa, EM/Gly: 247.17 ± 59.09 kPa, EM/DMSO 215.12 ± 116.99 kPa; Figure 4). In the EM/DMSO group, there was a significant reduction in tensile strength after storage for six months compared to the control group (0.46 ± 0.21 MPa vs. 0.87 ± 0.25 MPa, *p* = 0.02). The other values for tensile strength (after two months PBS: 0.59 ± 0.17 MPa, EM/gly: 0.63 ± 0.24 MPa, EM/DMSO: 0.73 ± 0.27 MPa; after six months PBS 0.51 ± 0.18 MPa, EM/gly: 0.58 ± 0.31 MPa, EM/DMSO: 0.46 ± 0.21 MPa) did not show statistically significant differences (all *p* ≥ 0.08). The stretching behavior of PDC was comparable between the groups (*p* ≥ 0.06).

### 3.4. Re-Epithelialization

**Figure 5:**
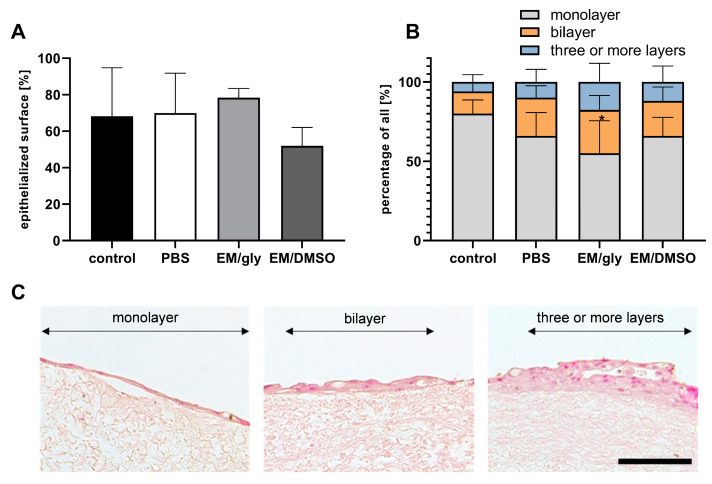
Recellularization capacity of fresh PDC (control) and PDC stored for six months in PBS (at 4 °C), in EM/gly (at −80 °C) and in EM/DMSO (at −196 °C) with primary HCEC. (**A**) Percentage of the epithelialized PDC surface after a 14-day cultivation period. (**B**) A subdivision of the grown epithelium (**C**) into monolayer, bilayer and three- or multi-layered epithelium. Representative images are shown in (C). * *p* = 0.01; n = 4; scale bar.

After cultivation for 14 days, epithelial cells adhered to PDC in all storage variants. The mean values of the epithelialized surface within the total length of the analyzed PDC sections ranged from 51.94 ± 8.76% in the EM/DMSO group to 78.34 ± 4.42% in the EM/gly group (control: 68.22 ± 23.03%, PBS: 69.94 ± 18.90%; Figure 5). Statistically, there were no significant differences in the epithelialized surface of PDC between the groups (*p* ≥ 0.35). In addition to the extent of re-epithelialization, epithelial stratification enabled a more precise characterization of the developed epithelia (Figure 5). In all groups, epithelium was dominantly single-layered (control: 80.03 ± 7.53%, PBS: 66.03 ± 12.77%, EM/gly: 55.04 ± 17.76%, EM/DMSO: 65.93 ± 10.18%), followed by two-layered (control: 14.00 ± 4.58%, PBS: 24.13 ± 6.50%, EM/gly: 27.29 ± 7.94%, EM/DMSO: 22.05 ± 7.69%) and finally three- or multi-layered epithelium (control: 5.98 ± 3.96%, PBS: 9.85 ± 6.87%, EM/gly: 17.67 ± 10.20%, EM/DMSO: 12.02 ± 8.75%). Only the PDC of the EM/gly group showed a slightly lower percentage of monolayered epithelium compared to the control group (*p* = 0.01); however, the percentage of bi- and multi-layered epithelium was not significantly different compared to the other groups (*p* ≥ 0.1).

## 4. Discussion

Previous studies indicated a high stability and biocompatibility of PDC, making it a potential alternative tissue for conjunctival reconstruction [22,23]. A simplified clinical use requires storage options and off-the-shelf availability of this tissue. As there are no studies on long-term storage of PDC to date, we analyzed the effect of three different storage methods for up to six months on its structural, mechanical and biological properties. The storage conditions chosen for this study represent commonly used techniques for tissue banking and were evaluated in several studies addressing the storage of decellularized tissues [24,26,27,31,32].

Histology and TEM showed that ECM architecture, fiber density, and fiber diameter of stored PDC were comparable to that of the control group. This is remarkable, as several publications have demonstrated that storage in PBS at 4°C for six months or more can lead to loosening of ECM structures [26,27,31], making it usually a short-term storage option [34]. In our study, also ultrastructurally, there was no evidence of fiber disruption or detachment of collagen structures from the fiber bundles as was found e.g. on decellularized human trachea after storage in PBS for twelve months [31]. A possible explanation may be the different structure and protein composition of PDC compared to trachea or other tissues studied such as esophagus and gonads.

Cryopreservation of PDC is a method of interest because it is known that freezing and thawing processes can have detrimental effects on the arrangement and integrity of collagen structures in tissues with a high collagen content [35,36]. This has been attributed to the repeated formation of ice crystals in the intracellular and extracellular space [35]. Cryoprotectants are used in order to reduce tissue damage as they prevent crystallization of water during freezing and were also used in this study [25]. Histological examination revealed that frozen and cryopreserved PDC showed structural preservation after six months of storage. Our finding is consistent with other studies on cryopreservation of decellularized tissues [26,27,37]. In addition to the analysis of ECM morphology, protein quantification did not show significantly decreased collagen and elastin amount within all storage methods. In the literature, there are different observations with regard to protein retention of various decellularized tissues after storage. While Baiguera et al. [31] and Gharenaz et al. [27] reported damage and a decreased protein amount for decelluarized human trachea and mouse testis after storage in saline for six and 12 months, a study by Wollmann et al. showed no significant collagen and elastin reduction in decellularized human heart valves after 18 months saline storage [38]. With regard to cryopreservation, we confirm the findings of others that protein within an acellular matrix is well preserved [26,27].

Changes in structural and biochemical components of PDC after storage can reduce ECM integrity and biomechanical properties. However, preservation of these characteristics is important for clinical application of PDC, as such an implant has to tolerate surgical handling and mechanical stress caused by globe motility [4]. Changes in the biomechanical properties of acellular matrices after longer storage were seen in studies on animal and human tissue [26,31]. Urbani et al. demonstrated a significant reduction in tensile strength in decellularized rabbit esophagus after storage in PBS (4 °C) for six months while no difference was seen in a storage method comparable to our EM/DMSO storage. Moreover, elasticity increased significantly after six months for both forms of storage [26]. In a study by Baiguera et al. on the storage of decellularized human tracheas in PBS over a one-year period, no significant reduction in tensile strength was seen. In our study, all PDCs were stretchable and tear-resistant. Only the tensile strength of the EM/DMSO group after storage for six months was significantly reduced compared to the control. However, neither histological or ultrastructural analysis nor protein quantification provided any structural changes that correlated with this result. Since PDCs of the EM/DMSO group showed the highest tensile strength after two months of storage, the significant reduction after six months should be interpreted with caution.

However, the relevance of the exact storage medium composition in the context of freezing has been questioned by several authors [39,40]. Poornejad et al. showed that freezing/thawing process without cryoprotectant led to biomechanical changes of native but not decellularized porcine renal tissue. In another study comparing storage methods of amniotic membrane, Wagner et al. found no significant biomechanical differences after freezing amniotic membrane with or without glycerol-containing medium, but described a change in biomechanics after a storage for more than six months, regardless of the storage medium [40]. Whether storage longer than six months has an effect on PDC needs to be investigated in further studies.

As various diseases of the ocular surface can be associated with damage or loss of conjunctival epithelial stem cells [41,42], many studies on tissues for ocular surface reconstruction deal with the recellularization of these matrices [23,43,44,45]. We were able to show that none of the storage conditions led to significant limitations regarding HCEC adhesion, viability and proliferation as all PDCs enabled re-epithelization. However, mucin-filled structures typical for goblet cells were not detected within the newly formed epithelium. Goblet cells are of great importance for the physiology of healthy conjunctiva and conjunctival replacement tissues, but their ability to be cultivated in vitro is limited [46]. In another study of our group, goblet cell formation was not observed up to a maximum period of 24 days when seeding an HCEC suspension previously co-cultured with a 3T3 fibroblast feeder layer onto PDC [23]. In the aforementioned study, though, the formation of goblet cells was observed from day 14 when a piece of native human conjunctiva was cultured directly on PDC (“explant method”). These results suggest that both the cultivation time and the niche environment have a significant influence on the formation of goblet cells, and that the explant method could be a legitimate option for further studies using PDC.

For completeness, it is worth mentioning that there are other storage forms described for acellular tissues not investigated in the current study, such as vitrification [28,47] or freeze drying [28,48,49]. Freeze drying is of interest because it allows storage at room temperature and thus, saves storage costs, enables easy transportation and does not require potentially toxic protective agents [24]. During freeze drying water is removed from frozen material under protection of substances such as sucrose or trehalose [50]. There are tissues that can be better preserved by freeze drying than by cryopreservation, for example, Zouhair et al. described that freeze drying did not alter the biomechanical properties of decellularized bovine pericardium while cryopreservation did [28]. However, data on usage of this method for acellular tissues are generally sparse and some studies reported damage to tissue architecture [24,51]. Therefore, specific requirements for the freeze dryer as well as further necessary chemical modifications of the process had to be found [28,51,52] and a tissue-dependent evaluation, appropriate laboratory equipment and experience with this method are required for safe use.

In conclusion, considering economic criteria and the practicability of the methods investigated in the current study, PBS at 4 °C may represent a preferable storage option for PDC. Refrigerators are commonly present in tissue banks and cause low costs compared to other forms of storage. In contrast, the use of liquid nitrogen and ultralow-temperature freezers are comparatively expensive and energy-consuming cryopreservation methods that are not always available. Moreover, a guideline from the Council of Europe (Guide to the quality and safety of tissues and cells for human application) expressed concerns about DMSO for tissue storage as it suggested not to use it as cryopreservant for amniotic membrane because of its cell toxicity [53]. Although we could not demonstrate a significant reduction in cell viability of HCEC cultured on PDC stored in EM/DMSO after washing procedure, in case of storage in PBS, the addition of cryoprotectants is not necessary. Therefore, additional time-consuming washing steps and potential biochemical influences of these substances on cell viability and proliferation can be avoided. Altogether, these findings may facilitate storage and potential clinical usage of PDC.

## Figures and Tables

**Table 1:** Specifications of materials, chemicals and instruments.

Product	Specification	Supplier	Supplier’s Location
Phosphate-buffered saline solution	14190250	Thermo Fisher Scientific	Waltham, MA, USA
Penicillin/Streptomycin	P4333	Sigma-Aldrich	St. Louis, MO, USA
Glycerol		Central Pharmacy University Hospital Duesseldorf	Duesseldorf, Germany
Dulbecco’s Modified Eagle’s Medium/ F-12 Ham	D8062	Sigma-Aldrich	St. Louis, MO, USA
Fetal bovine serum	S 0615	Bio-Sell	Feucht, Germany
Hydrocortisone	H0888	Sigma-Aldrich	St. Louis, MO, USA
Cholera toxin	C8052	Sigma-Aldrich	St. Louis, MO, USA
Sodium bicarbonate	25080060	Thermo Fisher Scientific	Waltham, MA, USA
Triiodo-L-thyronine	T6397	Sigma-Aldrich	St. Louis, MO, USA
Transferrin	T8158	Sigma-Aldrich	St. Louis, MO, USA
Adenine	A2786	Sigma-Aldrich	St. Louis, MO, USA
Insulin	I9278	Sigma-Aldrich	St. Louis, MO, USA
EGF	PHG0313	Thermo Fisher Scientific	Waltham, MA, USA
Freezing container	C1562	Sigma-Aldrich	St. Louis, MO, USA
Paraformaldehyde Roti^®^Histofix	P087.1	Carl Roth	Karlsruhe, Germany
Rotary microtome	RM2255	Leica Biosystems	Nussloch, Germany
Sircol™ Insoluble Collagen assay kit	S2000	Biocolor	Northern Ireland, UK
Fastin™ Elastin assay kit	F2000	Biocolor	Northern Ireland, UK
Material testing machine	zwickiLine Z0.5 TN	ZwickRoell	Ulm, Germany
Cell crowns	cellcrown™24	Scaffdex	Tampere, Finland
Paraffin	Surgipath Paraplast Plus	Leica Biosystems	Nussloch, Germany
Glutaraldehyde 25% in water	23114	SERVA Electrophoresis GmbH	Heidelberg, Germany
Cacodylic acid.Na-salt.3H2O	15540	SERVA Electrophoresis GmbH	Heidelberg, Germany
Phosphotungstic acid hydrate	P4006	Sigma-Aldrich	St. Louis, MO, USA
Spurr Embedding Medium ERL-4221D	21041	SERVA Electrophoresis GmbH	Heidelberg, Germany
Ultramicrotome	Leica UC7	Leica Microsystems	Wetzlar, Germany
Transmission electron microscope	Hitachi H-7100	Hitachi High-Tech Corporation	Tokyo, Japan

**Table 2:** Overview of the groups and the respective storage conditions.

Group	Storage Condition [Temperature]
PBS	PBS [4 °C]
EM/gly	Conjunctival epithelial cell medium + glycerol 85% (1:1 *v*/*v*) [−80 °C]
EM/DMSO	Conjunctival epithelial cell medium + 10% FBS + 10% DMSO [−196 °C, liquid nitrogen]
Control	Fresh PDC (stored in PBS for a maximum of 96 h)

## Data Availability

The datasets generated during this study are made available from the corresponding author upon request.
